# Surviving a Critical Care Admission for COVID‐19: A Qualitative Study of Experiences and Unmet Needs During the Recovery Period

**DOI:** 10.1111/nicc.70368

**Published:** 2026-02-02

**Authors:** Mary Gemma Cherry, Stephen L. Brown, Alicia A. C. Waite, Peter L. Fisher, Ingeborg D. Welters, Brian W. Johnston, Andrew J. Boyle, Christina Jones, TRIC Network

**Affiliations:** ^1^ Department of Primary Care and Mental Health University of Liverpool Liverpool UK; ^2^ School of Psychology University of New England Armidale Australia; ^3^ Critical Care, Liverpool University Hospitals NHS Foundation Trust Liverpool UK; ^4^ Institute of Life Course and Medical Sciences University of Liverpool Liverpool UK; ^5^ Liverpool Centre for Cardiovascular Science University of Liverpool Liverpool UK; ^6^ Wellcome‐Wolfson Institute for Experimental Medicine, Queen's University Belfast Belfast UK; ^7^ Regional Intensive Care Unit, Royal Victoria Hospital Belfast UK; ^8^ ICUsteps Charity, Kemp House London UK

**Keywords:** COVID‐19, critical care, intensive care unit, qualitative, unmet need

## Abstract

**Background:**

Admission to an intensive care unit (ICU) places substantial physical, psychological and existential burden on patients, but little is known about the support needs of survivors of COVID‐19 critical care.

**Aim:**

To explore the experiences of survivors of COVID‐19 critical illness during their recovery phase, including perceptions of care received and support offered to them. Research questions were (i) what are patients' experiences during ICU admission and in the recovery period at a psychological, physical and functional level and (ii) what are participants' preferences for support during the post‐discharge period?

**Study Design:**

A qualitative study nested within a national multicentre longitudinal study examining the psychological impact of COVID‐19 critical care. Semi‐structured interviews were conducted with adult patients aged ≥ 18 years, treated for presumed/diagnosed COVID‐19 infection and survived to ICU discharge following an admission of ≥ 24 h. Interviews explored patients' ICU and post‐discharge experiences and preferences for post‐discharge support. Analysis drew on principles of the constant comparative method and interpretive reflexive thematic analysis.

**Findings:**

Fifteen participants completed interviews. Participants reported uncertainties and difficulties in adjusting to their illness, to ICU care and to post‐ICU discharge. Participants experienced extreme functional loss and knew their illnesses to be highly dangerous, yet did not appraise their symptoms as being severe. This dissonance was exacerbated by the dearth of knowledge regarding the effects and prognosis of COVID‐19 critical illness. Participants desired follow‐up support but more commonly spoke of wanting to negotiate a clear path towards recovery and being disappointed in some professionals' failures to provide this. Psychological symptoms emerged later in survivorship, but patients did not routinely access psychological support.

**Conclusions:**

More tailored and consistent post‐discharge support, using novel approaches, may standardise equity of care and address patients' needs.

**Relevance to Clinical Practice:**

Greater support for patients and staff is needed to facilitate understanding and acceptance of uncertainty related to unprecedented public health emergencies such as COVID‐19.

## Impact Statements

- What is known about the topic
  ○ Admission to an intensive care unit (ICU) places substantial physical, psychological and existential burden on patients.
  ○ Little is known about the experiences and needs of survivors of COVID‐19 critical illness in the United Kingdom.
- What this paper adds
  ○ Participants experienced extreme functional loss and knew their illnesses to be highly dangerous yet did not appraise their symptoms as being severe. This dissonance was exacerbated by the dearth of knowledge regarding the effects and prognosis of COVID‐19 critical illness.
  ○ Participants desired follow‐up support but more commonly spoke of wanting to negotiate a clear path towards recovery and being disappointed in some professionals' failures to provide this. Psychological symptoms emerged later in survivorship, but patients did not routinely access psychological support.
  ○ More tailored and consistent post‐discharge support, using novel approaches, may standardise equity of care and address patients' needs.

## 1 Introduction

Admission to an intensive care unit (ICU) places substantial physical, psychological and existential burden on patients. The umbrella term ‘Post Intensive Care Syndrome’ (PICS) refers to the psychological, physical and cognitive sequelae of an ICU admission, including anxiety, depression, post‐traumatic stress, muscle weakness and long‐term cognitive impairment [1]. These symptoms are common across ICU survivor populations, irrespective of underlying diagnosis and can persist for months or years after discharge [1, 2]. Main risk factors for PICS include delirium, prolonged mechanical ventilation, deep or prolonged sedation (especially benzodiazepines), severe critical illness, older age, pre‐existing cognitive or mental health issues and prolonged immobilisation during the ICU stay [3].

Survivors of COVID‐19 critical illness experience many of the same challenges described in the wider PICS literature [4, 5, 6, 7], but the context of the pandemic is likely to have intensified or modified certain aspects of survivorship. For example, prolonged isolation, visiting restrictions, staff wearing personal protective equipment (PPE) and the lack of public knowledge of the condition may have heightened fear, disorientation and existential threat. Additionally, COVID‐19‐specific pathophysiology (e.g., prolonged ventilation, severe hypoxaemia, thrombotic complications) has been associated with significant physical and cognitive burden in recovery, suggesting that COVID‐19 survivors' experiences may differ from other ICU survivors' [8]. In recognition, the National Institute for Health and Care Excellence (NICE) highlights the need for comprehensive, multidisciplinary post‐discharge care for COVID‐19 survivors [9]. However, in a national survey evaluating the provision of follow‐up services in the United Kingdom for adult survivors of COVID‐19, only 42.1% included a clinical psychologist [10], reinforcing ongoing variability in access to essential support.

### 1.1 Background/Justification for Study

Learning from the experiences of patients across the wider ICU survivorship literature is fundamental to designing effective and equitable follow‐up care. However, given the unique context of the COVID‐19 pandemic, it is important to understand whether COVID‐19 survivors experienced ICU care and recovery in ways that diverge from, or align with, established patterns described in non‐COVID populations.

Although extensive research has examined ICU survivorship more broadly, relatively few qualitative studies have explored the experiences of survivors of COVID‐19 critical illness [11, 12, 13, 14, 15, 16]. The observed heterogeneity in psychological experiences among survivors of COVID‐19 critical illness has been partly attributed to the influence of cultural and contextual influences, including differences in family presence policies, staffing pressures and access to rehabilitation [17, 18]. Within the United Kingdom, only two qualitative studies have been published; one explored the experiences of older adults and their families, including families of deceased patients, regarding escalation to ICU [19]. The other reported the recovery experiences of six patients, highlighting the challenges of living with guilt, uncertainty and unexpected symptoms [20]. While the nature of post‐discharge support must ultimately be tailored to the individual needs of all ICU survivors, there remains limited understanding of how survivors of COVID‐19 critical illness in the United Kingdom experienced both their ICU stay and the subsequent recovery period. This gap hampers efforts to develop post‐ICU pathways that integrate lessons from the pandemic with the wider evidence base on PICS.

### 1.2 Aims and Research Questions

Our aim was to explore the experiences of survivors of COVID‐19 critical illness during their recovery phase, including perceptions of care received and support offered to them. Research questions were (i) what are patients' experiences during ICU admission and in the recovery period at a psychological, physical and functional level and (ii) what are participants' preferences for support during the post‐discharge period?

## 2 Design and Methods

### 2.1 Design

A qualitative design, using semi‐structured interviews nested within a national multicentre longitudinal study examining the psychological impact of ICU admission for COVID‐19 (the Psychological Impact of COVID‐19 on Intensive Care Survivors [PIM‐COVID] study), was used [21]. The ontological and epistemological stance adopted was underpinned by a social constructionist framework [22], which assumes that individuals construct meaning through their interactions with others and through their social and contextual environments. This perspective emphasises how people interpret and make sense of their experiences, making it well suited to research questions focused on how ICU survivors experience their ICU admission and recovery.

### 2.2 Setting and Sample

Participants were a subsample of patients who had been recruited to the PIM‐COVID main study (adult patients aged ≥ 18 years who survived to intensive care/high dependency unit discharge following an admission of ≥ 24 h and who were treated for presumed/diagnosed COVID‐19 infection) [21]. Participants were recruited through the 52 ICUs across England that participated in the multicentre trial (see Supporting Information in Waite et al. [7] for additional information regarding ICU sites).

### 2.3 Data Collection Tools and Methods

All eligible patients were given an information sheet outlining the main PIM‐COVID study, inviting them to complete questionnaires at 3, 6 and/or 12 months following discharge from ICU. Prior to patient contact, electronic healthcare records were accessed, and/or contact made with the patient's registered general practitioner (GP), to confirm the patient's survival status. Participants were recruited to the qualitative sub‐study at multiple timepoints (namely, after completing each set of questionnaires). Consenting participants were approached by phone or email and invited to participate in an interview to provide further information about their ICU and discharge experiences. A written patient information sheet was provided by email or post, following which a date and time were arranged for the interview to be conducted. Consent was provided by a returned consent form. Demographic data were collected prior to interview. A semi‐structured interview schedule, using open questions, prompts and reflection, was used to facilitate patients' talk. Closed questions were used to probe specific points. The pacing, sequencing and length of interviews were set by participants. The interviewer followed a topic guide but also pursued relevant participant‐generated ideas absent from the list. Topics included

- physical and psychological consequences patients may have faced during their recovery phase, including physical sequelae, such as loss of smell, breathlessness, fatigue and psychological issues such as isolation, anxiety, flashbacks or sleeping problems
- perceptions of follow‐up services offered by their GPs, the hospital and patient support groups
- engagement (if any) with services offered and reasons for this
- beneficial aspects of available support
- perceptions of how support could have been improved.

Further elements were added to the topic list as interviews unfolded. Interviews were conducted by ACCW, a medically qualified research fellow trained in conducting qualitative interviews, over the telephone or online (via MS Teams), to ensure equitable access for study participants. Interviews were audio‐recorded, anonymised and transcribed. Recordings were held in accordance with EU General Data Protection Regulations (GDPR) and the UK Data Protection Act (2018).

## 3 Data Analysis

Analysis drew on principles of the constant comparative method and interpretive reflexive thematic analysis [23]. Three authors led a process of iterating between the developing analysis and new data, developing and testing the analysis by periodic discussion. The analysis was interpretive and considered both latent and manifest aspects of the data, thereby acknowledging both the explicit content of interviews and possible meaning ascribed to statements. Analysis progressed in parallel with recruitment and was guided by the concept of information power [24], which proposes that the adequacy of a qualitative sample depends on the richness and relevance of the data in relation to the study aim rather than the number of participants. Information power was assessed throughout data collection and analysis, considering the study aim, quality and depth of interview dialogue, epistemological stance, analytic method and the focused aim of the study. Systematic data coding was performed; exceptional case analysis was discussed within the research team; and data were triangulated with quantitative data from the PIM‐COVID study to enrich findings and interpretation. Key findings are illustrated by italicised quotes attributed to individuals (participant numbers in parenthesis), with ellipses (…) indicating omitted text and explanatory comments in square brackets.

### 3.1 Ethics Statement

Ethical approval was received from the East Midlands‐Derby Research Ethics Committee: 20/EM/0247, in November 2020.

## 4 Findings

Invitations to participate in interviews were extended to 62 participants. Of these, 46 participants did not respond to email or phone contact, 16 consented including one participant who consented but did not reply to further contact to arrange an interview. As such, our sample comprised 15 patients (eight females and seven males). Twelve (80%) identified as White British. Mean length of ICU stay was 19.8 days (range 2–63 days). Median time between ICU discharge and interview was 16 months (IQR 13–24 months). Interviews had a mean duration of 70 min (SD ±9.7 min, range 49–88 min). Participants' clinical and socio‐demographic characteristics are shown in Table 1.

**TABLE 1:** Clinical and socio‐demographic participant characteristics.

Participant number	Age (years)	Sex	Ethnicity	Employment status	Highest educational achievement	Date of discharge from ICU	Length of ICU stay (days)	Index of multiple deprivation decile
1	35–39	Female	South Asian	Employed full‐time	Other qualification	April 2021	63	9
2	45–49	Male	South Asian	Employed full‐time	Degree or equivalent	April 2021	60	5
3	60–64	Female	White	Employed full‐time	Higher education (e.g., BTEC, NVQ, diploma, etc.)	May 2021	46	10
4	30–34	Female	White	Employed full‐time	Degree or equivalent	July 2021	5	1
5	70–74	Male	White	On sick leave	GCSE grades A*‐C or equivalent	August 2021	7	5
6	55–59	Male	White	Employed full‐time	Other qualification	August 2021	2	6
7	50–54	Male	White	Retired	Higher education (e.g., BTEC, NVQ, diploma, etc.)	May 2021	27	4
8	35–39	Female	White	Not provided	Not provided	August 2021	14	4
9	60–64	Female	White	Not provided	Not provided	August 2021	10	4
10	65–69	Male	White	Employed full‐time	Higher education (e.g., BTEC, NVQ, diploma, etc.)	July 2021	12	2
11	55–59	Female	White	Carer	GCE A level or equivalent	July 2021	7	2
12	50–54	Female	White	Employed full‐time	Degree or equivalent	August 2021	5	4
13	55–59	Male	White	Employed full‐time	GCSE grades A*‐C or equivalent	August 2021	3	6
14	70–74	Male	White	Retired	Other qualification	August 2021	30	4
15	30–34	Female	Black—Caribbean	Employed full‐time	Higher education (e.g., BTEC, NVQ, diploma, etc.)	August 2021	6	4

Abbreviations: A level, advanced level; BTEC, Business and Technology Education Council; GCE, General Certificate of Education; NVQ, National Vocational Qualification (all UK former or current educational qualifications).

Results are organised into a sequential account of patients' experiences, needs and preferences for support, identifying three over‐arching themes related to: (i) the ICU experience, (ii) the experience of step‐down care, post‐ICU discharge and (iii) the post‐hospital discharge experience. Within each theme, patients' experiences, behaviours, problems and preferences for support are outlined.

### 4.1 ‘I thought I was fine’: Navigating an Unexpected ICU Admission

#### 4.1.1 The Dissonance Between Participants' Perception of Symptoms and Their Health Status

Prior to hospital admission, patients did not generally report feeling subjectively unwell, yet found themselves fatigued, helpless and unable to perform simple self‐care functions. Indeed, seeking medical care often resulted from prompting and actions by others.

I said I felt fine, I explained I felt fine, I could taste things and I was quite happy because, as I say, it was the best I'd felt for however long. Anyway, my daughter wasn't satisfied with any of my excuses for being happy and in a good mood, so I phoned 999 and explained that I didn't appear to be myself. When the ambulance arrived, they put the oxygen monitor on me and it was only at 57%. So, they were saying that my euphoria state of being happy was hypoxia. And they 999'd me, blue lighted me straight into resus basically. All through this I still felt okay, I felt fine, I didn't feel ill. P12

[The GP] rang an ambulance up, which I were a bit shocked at. I'd have never rang ambulance up. I'd have gone, ‘I'm fine, I'll stay at home.’ So, when I went into hospital, I thought, ‘I'll be in a couple of days and I'll be back’. P7.

Amplifying participants' sense of dissonance between mild symptom experiences and their critical illnesses was their knowledge that COVID‐19 was something to be feared and that neither patients nor ICU staff were certain about the course and implications of critical COVID‐19.

I think the knowledge [of COVID] was just so minimal. And it still is to some degree, isn't it? (…) I think at the end of the day, we're, all of these medical professionals, as amazing as and as brilliant as they are, they're stabbing in the dark, aren't they? Because it is so new. It's not something we've really dealt with before, is it? P4

Participants treated with sedation and invasive ventilation described ICU as being a ‘surreal’ experience of temporal, existential, physical and mental disorientation, composed of hallucinations, gaps and contradictions triggered by their infection, anaesthetic and sedative drugs.

The nurses and doctors were amazing, but I was quite, I think I was quite difficult because I didn't believe them. You know, when they said, ‘Oh, your family are okay.’ I was, you know, I wasn't believing them. And then at one point, I started hallucinating them. So they were in the room with me and I was waving to them. And I remember the nurse saying to me, ‘Oh,’ you know, ‘who are you waving to?’ And I said, ‘Oh, that's my family over there.’ And she said, ‘It's one o'clock in the morning. They're not there. You know, it's just the drugs that were giving you and the oxygen. You know, you haven't got enough oxygen in your brain, so you're hallucinating, you know, what you want to see.’ P8.

The first thing I can remember [when I woke in ITU] is looking at the clock, thinking, ‘The clock's wrong’, because it had a date on the clock. And thinking, ‘Nah, that clock's wrong’, because obviously it's 11 days had passed. P6.

#### 4.1.2 The Process of Resolving Uncertainty

Participants found it challenging to deal with dissonance and uncertainty, which resulted in distress. To deal with this, ICU participants attempted to ‘anchor’ themselves by developing a better understanding of their situations. They did this in three broad ways. First, they desired and sought information but spoke about the difficulty in having no‐one other than clinical staff to explain or describe their situations or ‘plug the gaps’ in their memories (visitors being prohibited).

When I came out of the coma (…) I couldn't understand why my family weren't there. You know why my husband, where my husband was, where my daughter was, because I have an older daughter and where my mum was because I knew, you know, if I'm in hospital, if I'm that poorly in intensive care, my family would be here. They'd be sat by the bed. And I didn't really have any consciousness around you know what COVID was or anything. I'd sort of lost all that. So I was very distressed. P8

Second, they made comparison with others, particularly with seriously unwell or dying patients. For some, comparisons consolidated the reality that their illness was indeed serious, but also provided proportion, in that they were less unwell and still alive.

Some of the things that they [staff] were dealing with were far worse. Do you know what I mean? I mean, you'd hear people being rushed through, you know, and tracheas [sic] being put in within seconds of them being on the ward and stuff. And you think, you know, and no one was telling me these things, but you can hear it in in the other rooms and things cause it's only curtains that really separate the rooms. And you can, you can hear the alarms when all the nurses need to go running and all of these things. I thought, this is, I'm being silly here. P4

However, such comparisons could be a source of negativity. Participants expressed anger and frustration towards other patients at times, while also speaking of the stark reminders provided by other patients of their own poor health and mortality.

You do that sort of comparison [with other patients]. And some weren't coping. Others were very needy. Others wanted special diets where, I'm not eating the hospital food. My wife or my family's cooking it ‘cause we have a, not a special diet, but we have home cooked food, which is what I'm used to.’ And the wards accommodated it. But of course, the, it was not terribly fair on those coming round to clean up after them where you've got takeaways and all the sort of paraphernalia and there's Coke bottles and you think, ‘It's not their job to clear up after you because you feel so needy.’ P5

Some people were really, you know, putting them [staff] through their paces and really kind of treating them awfully and they take it with such patience and grace. It's incredible. Like, I can't thank them enough for being as amazing as they are. P4

Third, participants dealt with uncertainty regarding their prognosis through short‐term goal setting (such as independently using the toilet), including setting goals related to ICU discharge. This allowed them to gain a sense of improvement from small steps and reduced momentary distress.

You set minor goals, like not getting the commode in and things like that. Mentally, at this point I mean, that's all I was focused on, just the little things, little improvements and like if I drop something off the bed, actually getting to pick it up. And recovering it and things like that, it's just little things like that just kept you going. P2

#### 4.1.3 The Importance of Relationships and Communication

While in ICU, contact with familiar people was of great importance to survivors, in the absence of visits from relatives and friends; gratitude was expressed to staff who ‘bent rules’ to facilitate this.

[Staff] would take their glove off and they would just hold my hand and that skin contact was so important. You know, feeling someone's warmth and erm…. You know, they filled that void, didn't they, in the physical sense, where… the one that would have been filled by your friends or family. They did that and they were completely aware of it. They were sympathetic towards it as well. P1

All expressed gratitude to ICU staff for the care that they received, often citing religion or spirituality as important aspects of the care they received. For some, gratitude was shaped by comparison between the working conditions of healthcare staff and the guidance given to the general public to stay at home.

[An ICU nurse] prayed for me and that was a massive, that were really personal, do you know what I mean, it were really big for me? I had him a few nights and it was, it was just having him and just having that connection with someone that was, just little things like that. Those points when I thought I was sinking, I can honestly say without a shadow of a doubt, God sent someone to lift me out of, out of that mire, out of that place. P7

[There was a] nurse that I had sort of seen you know from the distance and she wasn't actually my one‐to‐one at the time but she did come over, sort of probably about an hour before the end of her shift. And she said to me, she says, ‘Don't worry.’ She says, ‘God will look after you. You know, you will be alright.’ And I mean I haven't been to church for a long, long time, but I didn't (…) She just said, ‘God will look after you and He'll make sure that you are alright.’ And we prayed. You know, we said you know the Lord's Prayer. She prayed for me. And it was so moving and after that, I did sort of feel, sort of like probably not a healing but I did sort of feel like a lot of weight and worry had left off and I could see, sort of, I could look forward. P3

### 4.2 Experiences of Transitioning to From ICU to Step‐Down Care

All patients initially welcomed the move from ICU to step‐down ward care, seeing this as a step towards regaining ‘normality’. However, most described the reality of transitioning away from the intensive, consistent and containing ICU environment as unexpectedly difficult.

It was quite unnerving actually, I think. I felt reassured when I had a nurse there 24/7. Whereas when I went up to the ward. I'll be like, you know, if I felt ill or you know, I needed quite a bit of anti‐sickness, you know and having to wait. I was like, ‘Oh, you know, what's wrong.’ But also again, I didn't want to make a burden of myself. I know they're really, really busy, you know, so. Yeah. That was tough. P8

Vulnerable and physically dependent on staff, patients described post‐ICU stepdown care experiences, characterised by feelings of powerlessness and loss of dignity co‐existing alongside gratitude for their survival and care received.

That first night, after I was transferred, was absolutely horrendous, the night into the next morning. It was something to do with… They had to take out the central line out of my neck because, they said, they're not trained to deal with that on the ward. So, they had to put a couple of cannulas in my arms (…) I don't know what happened but something happened with one of these cannulas and I was in absolute agony. I've never been‐ I'm not soft, by any means, but I've never been in as much pain in all my life. I was ready to rip these cannulas out of myself. I kept ringing the bell and people weren't coming. When they did come it was, I don't know, a healthcare assistant or somebody who wasn't qualified to take these things out. I just kept getting told all day, ‘[They're] in the handover [meeting]. Somebody will come soon.’ There was always a different reason why somebody hadn't been. P11

### 4.3 Navigating the Unexpected Landscape of Post‐Hospital Discharge Recovery

Post‐hospital discharge, patients were unprepared for the demands and trajectory of their recovery, viewing discharge from hospital as the end of their recovery.

You're delighted to come home but you've lost that very protective arm round you. That protective arm that's got all the experience, all the answers, that's got the access to the drug trolley for whatever medication you need. You miss that because all of a sudden, you're thinking, ‘Ah, haven't got this anymore.’ So, you just have to take things a little gingerly. P5

At interview, most participants reflected that they felt that they had been discharged from hospital too early. For some, this was the result of advocating or bargaining for discharge, either to avoid the hospital environment or to return home, whereas for others, they felt this was due to NHS pressures rather than in their best interests. Evident was a sense of abandonment and dissatisfaction with post‐ICU hospital care, which patients struggled to reconcile with their ICU experiences and societal pressures to be grateful to healthcare staff.

I don't feel I was well enough to come home when I did, if I'm honest (…) knowing what I know now about my recovery and how difficult it was at home, I would've probably pushed to stay a little bit longer, but you wouldn't believe that if you'd have heard me the last few days I was in there. P11

It was a big step, but it was one that I was desperate for. I really wanted to go home and in hindsight I should have probably stayed in hospital and recovered a bit more. I don't think I was ready to come out. P8

Other patients had high expectations of their recovery and felt fearful, disappointed and frustrated when they were not able to meet these. They described temporal support needs, with a desire for practical support to troubleshoot difficulties with toileting and breathing taking priority in the days following discharge.

The frightening bit started when I got out of hospital, trying to climb the stairs. What I've been left with is scarring of the lungs, due to the pneumonia, and breathlessness. Things have slightly improved, but I don't know whether it's me coping with my limitations these days. But it was frightening the first day home, and I climbed the stairs and I thought, ‘I can't go back down there and try it again.’ P10

Oh, God, it took ages for me to get my strength back. Ages. Ages. Ages. It was a long time. It was a long process. Because even when I came home, I was weak for ages. I couldn't come up and down the stairs. Even to go to the bathroom, I would be just out of breath. It was just awful. (…) I think sometimes I do feel like I get a little bit anxious, but I think it's because of the frustration as well of, you know, it taking so long. P15

A consequence of the earlier described disjunction between felt experience and inferential knowledge of their conditions was that participants feared that they could not judge their own medical progress.

How will I know if I'm poorly? If I didn't feel poorly then and I nearly died, how will I know that I'm seriously ill? So that gauge, that filter, has changed, because normally when you feel ill you feel ill don't you? P12

Participants desired and expected support from professionals, but often experienced this as inconsistent, recalling mistakes that hindered their ability to breathe or regain mobility immediately post‐discharge. Where support was offered, it was often remote or brief or delivered too late after discharge to be helpful.

I think it would have been really good to have had more contact afterwards and because I was struggling with lots of things. You know, I was struggling with being able to walk, but also just general, you know, not being able to lift things, feeling really, really weak and tired. And as the weeks went on, different things sort of cropped up, you know. So, I think ideally maybe a weekly check in and for someone to set me a goal. (…) P8

Physio was actually very disappointing. They came about three weeks after I got home. A very, very quick visit, he assessed my ability to walk and then basically they said, ‘Right, well, you can walk. Just keep walking. You'll be fine. You'll get better eventually.’ I'm being glib. That's not how they put it. They were very professional about it. But that's basically what I understood and I got discharged from the physio service. As far as they were concerned, if I was mobile and just kept being mobile, I'd get better over time. P2

In the absence of formal support, patients relied on family and friends or tried to ‘get on with it’ regardless of whether this was appropriate for their stage of recovery. Patients who had supportive families found discharge a welcome relief; those that did not felt very alone, which resulted in or exacerbated mental health difficulties.

And my husband was such a good support. Thinking back now, I just, I don't know how we got through those sort of six weeks before I was able to sort of stand up and move around properly. P1

…my family are my support group. If I didn't have that, I don't think I would be… you know. My kids really, and my husband, they've been so, so supportive. They wouldn't let me do anything. If I'm going to get up to walk, they'll be there, ‘Mum, drink some water,’ because they'll tell me to breathe if I move, and afterwards if I can't, they are ‘Where's your water? Where's your water? Where's this?’ They really look after me. P15

Although fear of reinfection with COVID‐19 was present immediately post‐hospital discharge, participants' psychological needs generally emerged later in survivorship. Participants described intrusive memories of their ICU stay, difficulty in reconciling their experiences and disbelief or uncertainty about the severity of their illness. They described engaging in a range of maladaptive coping strategies such as rumination, avoidance and hypervigilance.

[Now], I go over the whole thing in my mind sometimes. It's like on a loop and I'll try and switch it off. I'll think about something else for maybe a minute, two minutes and then it sort of creeps back in. P8

I try not to [talk to family about how I feel], to be honest (…) I don't want them to be worrying about me all the time, so I tend to just probably bottle it up I suppose. I don't know. P11

Longer‐term, participants expressed both a want and a need for follow‐up support—in part, to address the psychological impact of ICU admission—but this was not consistently offered. Where it was offered, it was generally gratefully received, but the lack of consistent or appropriate support hindered recovery and invalidated some participants' experiences, resulting in guilt or doubt about the legitimacy of their ICU experience, the severity of their illness and therefore entitlement for follow‐up.

I didn't realise I'd had sepsis until I sort of read it in my discharge papers. (…) If somebody had just sort of come to see me and sort of say, ‘Have you got any questions?’ or something, you know, or just sort of go through the timeline of what happened, I'd have left you know with a bit of sort of clear in my head. P3

This is where I got into cynical mode, where you're thinking, ‘Alright, you're not the only one who's ill.’ So, you accept that, but at least you want some attention and acknowledgement of what you've been through. P10

Participants described difficulty in knowing who to ask for support, often viewing follow‐up as the remit of the GP or community services.

I mean, for me, intensive care is to deal with the initial issue isn't it really? It's to deal with the, it's to deal with the imminent problem, isn't it? Do you know what I mean? I mean, I think the kind of the follow up care should really come from your general practice really shouldn't it? P4

Once you're discharged from the sort of hospital you really now have to go through the GP. Erm. Because you're discharged from the hospital now, so in theory you're now outside their remit. P5

## 5 Discussion

Similar to patients pre‐pandemic [25, 26, 27] and to people with COVID‐19 in other international and UK studies [11, 12, 15, 16, 20], our participants described many difficulties that are well recognised across the wider ICU survivorship literature. These included uncertainty, challenges adjusting to ICU care, reduced functional capacity and the demands of step‐down care and discharge planning. In this sense, the core features of their recovery trajectory—fatigue, weakness, cognitive fog, emotional vulnerability and the need to ‘make sense’ of what had happened—are not specific to COVID‐19 but align with established PICS‐related experiences in survivors of prolonged ventilation and critical illness more generally [25, 27]. However, our data also point to how the pandemic context shaped these otherwise generic ICU experiences; participants reported a paradox in which they *knew* their illness to be highly dangerous—reinforced by pervasive public messaging, high levels of media attention and the global emergency—yet *did not feel* as unwell as the severity of their clinical course implied. This dissonance between symptoms and threat is not unique to COVID‐19, but, in this population, it was amplified by the unprecedented uncertainty surrounding a novel disease, rapidly changing clinical knowledge and the absence of clear prognostic information. The lack of visitors, staff wearing PPE and limited opportunities for bedside communication further constrained the usual interpersonal scaffolding that often helps ICU survivors construct coherent narratives about their illness. These features, therefore, represent contextual rather than clinical differences.

Patients' needs can thus be seen in two interlocking dimensions: explanatory needs concerning dissonance and uncertainty in how COVID‐19 is experienced and support needs for the functional and psychological implications of the illness. Explanatory needs refer to patients' capacities to organise their experiences in a coherent way, enabling them to navigate pathways and set realistic recovery goals. The value to participants of these understandings was exemplified by their spontaneous information seeking, social comparison and goal‐setting attempts, both in hospital and after discharge. Support needs refer to patients' wishes for both emotional and practical help with recovery. While in hospital, healthcare professionals were a primary source of emotional support in the absence of visitors. Post‐discharge, patients sought support from both loved ones and formal healthcare services to aid their recovery. For some, clinical pathways specific to COVID‐19 facilitated earlier discharge home than usual for some patients, with access to oxygen monitoring devices and/or supplemental oxygen where required, with mixed reactions from patients we interviewed.

Participants' awareness of their needs emerged slowly, partly because they initially underestimated the severity of their illnesses. A large literature on self‐regulation [28] suggests that people interpret the severity and implications of illness inductively; that is, they view illness through the lens of bodily symptoms, such that severity is inferred through salient and debilitating bodily experiences. The dissonance between felt symptoms and their implications created two problems. First, despite a declarative understanding of the serious nature of COVID‐19, our participants initially possessed high expectations of recovery. This led to confusion and disappointment as they slowly adjusted to the severity of their illnesses and the difficult road to recovery. Second, dissonance and participants' understandable reluctance to request help from staff whom they felt to be overwhelmed meant that participants did not assertively press for information or follow‐up services. Some patients' perceptions that they had been discharged home earlier than indicated may be reflective of functional weakness/dependence that became more obvious once at home. As several reported, services were not routinely offered meaning that participants did not receive services that they felt they needed.

Support needs were broadly consistent with other post‐ICU populations [29]. Participants wanted clear guidance on recovery, help with immobility and attention to emerging psychological symptoms such as fear of reinfection or post‐traumatic stress—findings that parallel high rates of psychological distress after critical illness more generally [7, 30]. The pandemic context, however, may have intensified these experiences through prolonged isolation, reduced rehabilitation opportunities and greater societal anxiety and uncertainty.

### 5.1 Implications for Practice and Further Research

Several implications can be proposed from these findings, particularly the findings that participants' understandings of their needs developed long after discharge. First, our findings highlight the need for ICU follow‐up models that support both generic PICS‐related recovery and the additional explanatory needs that arise when patients experience serious illness during a public health emergency. Providing accessible information about ICU events, typical recovery trajectories, and how to interpret persistent symptoms may help bridge the gap between patients' expectations and the reality of recovery. Although there has been an overall increase in ICU follow‐up clinic provision across the United Kingdom, there remains significant variation in the follow‐up provided. In keeping with prior literature [31], participants in our study wanted to be followed up or to receive follow‐up earlier than provided but did not know who to ask for help and experienced difficulties getting a GP appointment [32]. Similar barriers and uncertainties in post‐ICU follow‐up support have been reported by GPs [29]. The opportunity to provide timely support to patients may have been missed, and the potential impact on long‐term psychological and functional recovery is currently unknown. Further research should explore patient‐triggered remote physical and self‐guided psychological interventions, in addition to more traditional face‐to‐face, telephone or virtual support options. Novel approaches may provide an alternative to the ICU follow‐up clinic appointment or GP acting as a gateway to accessing support and may provide patients with a consistent and accessible means of resolving their explanatory and support needs. Preliminary evidence suggests that attendance at virtual or telephone follow‐up appointments is higher than at face‐to‐face appointments, lending support for this approach to maximise patient engagement and reduce patient and staff burden [33]. This should be in place for use in a subsequent public health emergency.

Second is the need to explicitly support both patients and staff to deal with the uncertainty that a public health crisis brings. Current guidance for psychological support post‐discharge does not mandate what it should look like, beyond stipulating that it should be delivered by health, clinical or counselling psychologists. However, psychological input for patients varies across the United Kingdom, with some receiving support in hospital, some upon discharge and others receiving none. Our findings suggest that an explicit focus on tolerance of uncertainty and resolution of disjunction between illness perceptions and functional limitations may be beneficial for patients. Staff may also benefit from structured intervention or support to communicate uncertainty to patients and families [34].

### 5.2 Limitations

A strength is recruitment of a national clinical sample with a range of demographic characteristics and experiences and elicitation of rich data pertaining to participants' experiences of ICU care and subsequent discharge. The sample of participants included in this sub‐study was similar to the wider PIM‐COVID study, where 89.3% of participants responding to questionnaires at 12 months were White. Due to the small sample size, the important inclusion of two South Asian and one Black participant has led to a slight higher representation of these minority groups compared to the overall study population (Interview sub‐study participant ethnicity: Asian 13.3%, Black 6.67% compared to the PIM‐COVID study where Asian participants accounted for 4.9% and Black 2.7% out of 1049 participants at 12 months) [7]. Limitations in the findings arise from two sources: difficulties and biases in patients' recall of their experiences and meanings that they drew from them and analysts' own interpretive biases. Many patients were interviewed a considerable time after the experiences that they described. Thus, it was difficult to disentangle their being unable to remember or interpret experiences (an important theme) from simple fragmentation of memory over time. The analytic team was composed of an ICU medical consultant and two psychologists. Possible bias arises from this narrow range of professional (non‐patient) experience. In particular, our previous quantitative research examined uncertainty, symptom appraisal and psychological distress—allowing insight into these phenomena in this study during theme generation but also representing a pre‐existing set of assumptions brought into the analytic process.

## 6 Conclusion

In summary, this study highlights the unique paradox experienced by COVID‐19 ICU survivors; the disjunction between perceived and actual functional status, compounded by limited knowledge of the course of COVID‐19. We identify two unmet functional and supportive care needs. More tailored and consistent post‐discharge support, using novel approaches, may standardise equity of care and address patients' needs, particularly in the case of a further public health emergency. Greater support for patients and staff is needed to facilitate understanding and acceptance of uncertainty related to unprecedented public health emergencies.

## Author Contributions

**Mary Gemma Cherry, Stephen L. Brown, Ingeborg D. Welters, Alicia A. C. Waite, Peter L. Fisher:** conceptualization—Ideas; formulation or evolution of overarching research goals and aims. **Mary Gemma Cherry, Stephen L. Brown, Alicia A. C. Waite:** data curation—Management activities to annotate (produce metadata), scrub data and maintain research data (including software code, where it is necessary for interpreting the data itself) for initial use and later re‐use. **Mary Gemma Cherry, Stephen L. Brown, Alicia A. C. Waite:** formal analysis—Application of statistical, mathematical, computational or other formal techniques to analyse or synthesise study data. **Alicia A. C. Waite, Ingeborg D. Welters, Mary Gemma Cherry, Andrew J. Boyle, Brian W. Johnston:** funding acquisition—Acquisition of the financial support for the project leading to this publication. **Alicia A. C. Waite, Mary Gemma Cherry, Stephen L. Brown:** investigation—Conducting a research and investigation process, specifically performing the experiments or data/evidence collection. **Mary Gemma Cherry, Stephen L. Brown, Peter L. Fisher:** methodology—Development or design of methodology; creation of models. **Alicia A. C. Waite, Brian W. Johnston, Andrew J. Boyle:** project administration—Management and coordination responsibility for the research activity planning and execution. **Mary Gemma Cherry, Stephen L. Brown, Alicia A. C. Waite, Ingeborg D. Welters:** supervision—Oversight and leadership responsibility for the research activity planning and execution, including mentorship external to the core team. **Mary Gemma Cherry, Stephen L. Brown, Alicia A. C. Waite:** visualisation—Preparation, creation and/or presentation of the published work, specifically visualisation/data presentation. **Mary Gemma Cherry [lead], Stephen L. Brown, Alicia A. C. Waite:** writing – original draft—Preparation, creation and/or presentation of the published work, specifically writing the initial draft (including substantive translation). All authors writing – review and editing—Preparation, creation and/or presentation of the published work by those from the original research group, specifically critical review, commentary or revision—including pre‐ or post‐publication stages.

## Funding

This work was supported by the Intensive Care Society (ICS) Young Investigators Award. Additional charitable funding was provided by the Mersey School of Anaesthesia (MSA) on application.

## Ethics Statement

Ethical approval was received from the East Midlands‐Derby Research Ethics Committee: 20/EM/0247.

## Consent

Informed consent was obtained.

## Conflicts of Interest

The authors declare no conflicts of interest.

## Data Availability

The data that support the findings of this study are available on request from the corresponding author. The data are not publicly available due to privacy or ethical restrictions.
